# Reliability of the Clinical Frailty Scale in very elderly ICU patients: a prospective European study

**DOI:** 10.1186/s13613-021-00815-7

**Published:** 2021-02-03

**Authors:** Hans Flaatten, Bertrand Guidet, Finn H. Andersen, Antonio Artigas, Maurizio Cecconi, Ariane Boumendil, Muhammed Elhadi, Jesper Fjølner, Michael Joannidis, Christian Jung, Susannah Leaver, Brian Marsh, Rui Moreno, Sandra Oeyen, Yuriy Nalapko, Joerg C. Schefold, Wojciech Szczeklik, Sten Walther, Ximena Watson, Tilemachos Zafeiridis, Dylan W. de Lange, the VIP2 Study Group

**Affiliations:** 1 Department of Anaesthesia and Intensive Care, Dep of Clinical Medicine, Haukeland University Hospital Bergen Norway, University of Bergen, 5019 Bergen, Norway; 2 Sorbonne Université, INSERM, Institut Pierre Louis D’Epidémiologie Et de Santé Publique, Saint Antoine Hospital, AP-HP, Hôpital Saint-Antoine, Service de Réanimation, 75012 Paris, France; 3 Department of Anaesthesia and Intensive Care, Ålesund Hospital, Ålesund, Norway; 4 Dep of Circulation and Medical Imaging, NTNU, Trondheim, Norway; 5 Deparment of Intensive Care Medicine, CIBER Enfermedades Respiratorias, Corporacion Sanitaria Universitaria Parc Tauli, Autonomous University of Barcelona, Sabadell, Spain; 6 Department of Anesthesia and Intensive Care Medicine, Humanitas Clinical and Research Center–IRCCS, Via Alessandro Manzoni 56, 20089 Rozzano, MI Italy; 7 AP-HP, Hôpital Saint-Antoine, Service de Réanimation, 75012 Paris, France; 8 Faculty of Medicine, University of Tripoli, Tripoli, Libya; 9 Department of Intensive Care, Aarhus University Hospital, Aarhus, Denmark; 10 Division of Intensive Care and Emergency Medicine, Department of Internal Medicine, Medical University Innsbruck, Innsbruck, Austria; 11 Division of Cardiology, Pulmonology and Vascular Medicine, University Hospital Düsseldorf, Heinrich-Heine- University, Düsseldorf, Germany; 12 Research Lead Critical Care Directorate St George’s Hospital, London, UK; 13 Mater Misericordiae University Hospital, Dublin, Ireland; 14 Faculdade de Ciências Médicas de Lisboa (Nova Médical School), Unidade de Cuidados Intensivos Neurocríticos E Trauma. Hospital de São José, Centro Hospitalar Universitário de Lisboa Central, Lisbon, Portugal; 15 Department of Intensive Care 1K12IC, Ghent University Hospital, Ghent, Belgium; 16 European Wellness International, ICU, Luhansk, Ukraine; 17 Department of Intensive Care Medicine, Inselspital, Universitätsspital, University of Bern, Bern, Switzerland; 18 Intensive Care and Perioperative Medicine Division, Jagiellonian University Medical College, Kraków, Poland; 19 Heart Center, Linkoping University Hospital, Linkoping, Sweden; 20 ICU, St George’s University Hospital, London, UK; 21 Intensive Care Unit General Hospital of Larissa, Larissa, Greece; 22 Department of Intensive Care Medicine, Dutch Poisons Information Center (DPIC), University Medical Center, University Utrecht, Utrecht, The Netherlands

**Keywords:** Clinical frailty scale, Inter-rater variability, Intensive care, Octogenarians

## Abstract

**Purpose:**

Frailty is a valuable predictor for outcome in elderly ICU patients, and has been suggested to be used in various decision-making processes prior to and during an ICU admission. There are many instruments developed to assess frailty, but few of them can be used in emergency situations. In this setting the clinical frailty scale (CFS) is frequently used. The present study is a sub-study within a larger outcome study of elderly ICU patients in Europe (the VIP-2 study) in order to document the reliability of the CFS.

**Materials and methods:**

From the VIP-2 study, 129 ICUs in 20 countries participated in this sub-study. The patients were acute admissions ≥ 80 years of age and frailty was assessed at admission by two independent observers using the CFS. Information was obtained from the patient, if not feasible, from the family/caregivers or from hospital files. The profession of the rater and source of data were recorded along with the score. Interrater variability was calculated using linear weighted kappa analysis.

**Results:**

1923 pairs of assessors were included and background data of patients were similar to the whole cohort (*n* = 3920). We found a very high inter-rater agreement (weighted kappa 0.86), also in subgroup analyses. The agreement when comparing information from family or hospital records was better than using only direct patient information, and pairs of raters from same profession performed better than from different professions.

**Conclusions:**

Overall, we documented a high reliability using CFS in this setting. This frailty score could be used more frequently in elderly ICU patients in order to create a more holistic and realistic impression of the patient´s condition prior to ICU admission.

## Background

Frailty assessment is increasingly used in critically ill elderly patients and has in many studies been shown to correlate with outcomes [1–3]. Frailty assessment has recently been suggested as one of several elements that could theoretically considered for decision to admit patients to the ICU during the present pandemic [4], although firm evidence for its use is lacking. Traditionally, frailty assessments are performed within the context of a comprehensive geriatric assessment and require active participation from the patient [5]. Understandably, this is not feasible in most acutely admitted or critical ill patients, and hence other methods have been developed to overcome this problem. One of the most frequently used tool for frailty assessments in this setting is the Clinical Frailty Scale (CFS) [6] developed from the large Canadian studies of frailty that established the cumulative deficit approach to frailty. The CFS has since increasingly been used in intensive care as well as in other emergency settings and was found in a recent systematic review to be the most frequent instrument used to assess frailty in ICU patients, but is only properly validated in patients ≥ 65 years [7].

As in any assessment method, the psychometric properties of the test are important. Regarding the CFS, the original publication [6] established construct validity by comparing it with the frailty index [8]. Inter-rater reliability of the CFS has been tested in a limited number of patients in three studies [9–11] and the feasibility was clinically demonstrated in the VIP1 study where CFS was collected in 99.8% of the 5187 patients included [1]. As a pre-defined sub-study nested within the VIP2 study [12] we additionally planned to perform a large international assessment of CFS reliability.

Interrater variability may vary for several reasons: individual differences in how to use the CFS, the rater's profession and experience, and the source of information necessary to perform a frailty score. Our hypothesis is that the CFS, being intuitive to perform, may vary little with the rater's profession and source of available information to perform the score.

The main aim of this study was to document inter-rater reliability within a large prospective observation study and assess the results of the score being derived from different raters and dissimilar information source, and in addition study potential variances between countries.

## Methods

### Study design and setting

The observational VIP-2 study was performed in acute ICU admissions of patients ≥ 80 years, and its primary aim was to describe the influence and interaction of several geriatric syndromes: frailty, co-morbidity, the activity of daily life, and cognition on many different outcomes. The study was performed over 12 months in 2018–2019 and included 3920 patients from 22 countries. More details and results can be found in the original publication [12]. Units could voluntarily sign up additionally to participate in a pre-defined sub-study of the inter-rater variability of CFS. The English version of CFS was used except for France and Switzerland using a validated version in French [13].

### Clinical Frailty Scale (CFS)

The CFS was used to assess frailty in all recruited patients as it presented prior to the acute event and admission to the ICU. The CFS is a pictographic scale from 1–9 describing nine different grades of frailty with a short text attached [6]. Patients with scores from 1 to 3 are considered not frail, 4 is pre-frail or vulnerable, and 5 to 9 are considered to be frail. No specific training, except a written explanation of the use of the CFS, was given to the participating units where many, but not all units had prior experience with using it.

### Assessment performed by different raters

In this study, two different study personnel from the ICU independently and blinded for each other results, assessed the patient at admission (first 24 h in the ICU) using the CFS with input from patients if possible, if not from care-givers or the medical and nursing hospital notes. The second rater was free to use sources of input and was not constrained to use the same as rater 1. The CFS score was noted for assessor 1 and 2 with information about the profession of the assessor: ICU nurse, ICU physician, dedicated study person, or other. Furthermore, they documented the kind of information that was used to perform the score. These data were then recorded in the electronic case record form (CRF) for the VIP-2 study by the local study investigator.

The assessors were named Rater 1 and Rater 2. In the analysis of data, the CFS rating was considered as an ordinal variable, and the occupation of the assessors were grouped as ICU nurse; ICU physician; research staff or other. The main source where the information was obtained was classified into 4 groups: (a) from the patient; (b) from family/care-givers; (c) from hospital records; and (d); another source, and they could only choose one option.

### Registration and ethics

This pre-defined sub-study was registered on Clinical Trials.gov identifier NCT03370692 at the same time as the main study. The main study was approved by ethical committees in all participating countries by institutional research boards, for details see the VIP-2 study main paper [12]. Since this study involved health professionals (raters) in some countries, this sub-study had to go through an independent review, and the rater then had to give informed consent to participate.

### Statistical analyses

A statistical analysis plan was discussed in the VIP2 study group and was decided to adopt to the guidelines for reporting of reliability and agreement studies (GRAAS) [14], see Additional file 1.

Data were analysed using SPSS version 25.0 (IBM, Armonk, NY USA) and with MedCalc 19.0 (http://www.medcalc.org Ostend, Belgium). The inter-rater reliability was assessed using linear weighted kappa in order to minimise outlier ratings and with intraclass correlation coefficient where raters for each subject were selected at random and with a one-way random effects model. We first analysed the inter-rater variability using all pairs then compared raters from different professions, information sources and participating countries. In the manuscript, we further use the accepted grouping of weighted kappa: Poor: 0–0.2, Fair (0.21–0.4), Moderate (0.41–0.6), good (0.61–0.8) and very good (0.81–1.0) [14, 15].

## Results

20 countries and 129 ICUs contributed to the inter-rater study that included 1923 pairs of raters, and hence two independent CFS assessments. This represented 49.1% of the whole VIP-2 study population, and patients’ details compared to patients not studied are given in Table 1.

**Table 1:** Details of VIP-2 patients studied compared to those not studied

	Included	Not included
Number	1923	1997
Age (median IQR) year	84 (81–87)	84 (81–87)
Male sex (%)	53.8%	53.3%
ICU LOS (median IQR) days	4 (1.96–8.89)	4 (1.71–7.12)
SOFA score (median IQR)	6 (4–9)	6 (4–9)
CFS fit (1–3)	789 (41%)	754 (38.1%)
CFS vulnerable (CFS 4)	407 (21.1%)	384 (19.4%)
CFS frail (CFS 5–9)	732 (37.8%)	840 (42.5%)
IQCODE (median, IQR)	3.25 (3–3.75)	3.19 (3–3.56)
Katz ADL score (mean)	4.8	
Co-morbidity/polypharmacy score (mean and 95% CI	10.6 (10.4–10.9)	
Admission groups (%)		
Acute respiratory failure	22.8	25.3
Emergency surgery	15.4	12.3
Sepsis	14.5	13.0
Acute circulatory failure	13.8	13.8
Respiratory and circulatory	10.7	12.2
Trauma	6.1	4.6
Cerebral failure	5.8	4.0
Other	10.4	13.0

The CFS is the value from Rater 1, and the % relates to the column (within the subgroup)

*SOFA* sequential organ failure assessment, *CFS* clinical frailty scale, *IQCODE* informant questionnaire on cognitive decline in the elderly, *ADL* activity of daily living, *CPS* Co-morbidity and Polypharmacy score

**Fig. 1:**
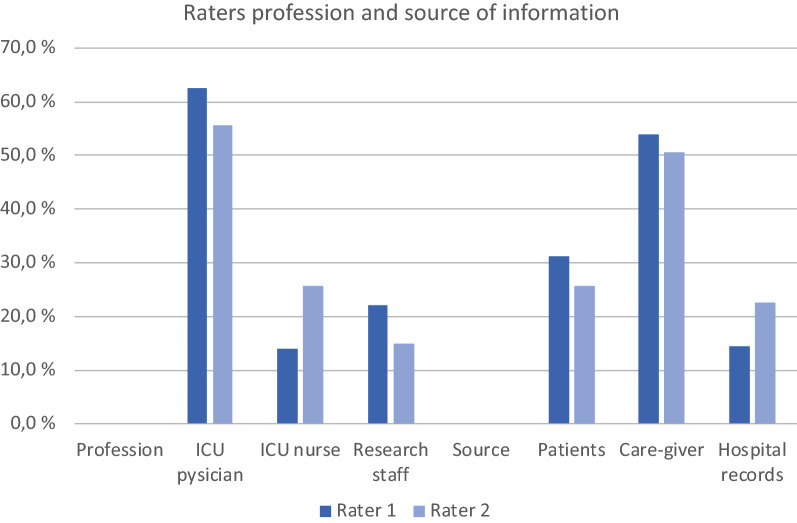
Raters profession and source of information in the two groups

Overall the number of completed CFS in the VIP-2 study was 99.6%, higher than activity of daily life score (ADL): 88.6% or cognition (IQCODE): 76.0%, showing very high compliance with this score. The profession of rater 1 and rater 2 were most often ICU physicians followed by ICU nurses, and the source of information for the rating was most often the family/care-givers (Fig. 1). The mean CFS from rater 1 and 2 was 4.18 (± 1,764) and 4.25 (± 1.76), respectively. Since the “other” group of raters and information sources were few and not specified, we have excluded these from further analysis.

The 9 different pairs of raters with regard to the profession are given in Table 2. The weighted kappa for all pairs was 0.86 (95% CI 0.84–0.87).

**Table 2:** Distribution of pairs of Rater 1 versus Rater 2

Profession of raters	Rater 1		
Rater 2^a^	ICU nurse	ICU physician	Research staff
ICU nurse	57	309	126
ICU physician	162	785	120
Research staff	46	96	138

^a^Missing pairs in 4 patients

The intraclass correlation coefficient (absolute agreement) was 0.93 for single measures and 0.96 for average measures, and the weighted kappa for all measures was 0.86 (95% CI 0.84–0.87) (Table 3). Worth noting is the distribution of scores of 4 and 5 in Table 3. A noteworthy number of rater one and two have scores above and below these values. Among rater one, 30 of 402 (7.4%) scored one or more CFS classes above 4 and by rater two in 65 of 407 (16%) patients demonstrating some difficulty of judging vulnerable from frail patients.

**Table 3:** Intraclass variance Rater 1 (CFS1) and Rater 2(CFS2); weighted kappa (linear) 0.86 (0.84–0.87)

CFS 2	CFS 1									
	1	2	3	4	5	6	7	8	9	Sum %
1	*76*	3	0	1	1	0	0	0	0	81 (3.5%)
2	0	*7*	1	0	0	0	0	0	0	196 (14.0%)
3	2	40	*403*	25	7	2	0	1	0	480 (25.0%)
4	1	4	54	*313*	22	7	1	0	0	402 (20.9%)
5	0	0	10	58	*163*	28	2	0	1	262 (13.6%)
6	0	0	5	5	43	*177*	17	2	0	249 (12.9%)
7	0	0	0	1	2	44	*126*	8	0	181 (9.4%)
8	0	0	0	1	0	3	13	*46*	2	65 (3.4%)
9	0	0	0	0	0	0	0	0	*7*	7 (0.4%)
Sum %	94 (4.9%)	202 (10.5%)	493 (25.6%)	407 (21.2%)	240 (12.5%)	261 (13.6%)	159 (8.3%)	57 (3.0%)	10 (0.5%)	1923

The results in Table 4 demonstrate the variability between pairs from different professions and less variability when similar source for information was used for both pairs. The best results were obtained when both raters were either nurses or physicians, and mixed pairs of assessors performed slightly worse. Likewise, there are better results when information does not come from the patients. There is also a good performance of the CFS across countries, but the three countries with the least number of pairs included performed less well than the others, although most countries were overall classified as very good (weighted kappa ≥ 0.80).

**Table 4:** Weighted kappa in subgroups (physicians and nurses) and 8 countries (≥ 100 pairs)

Group profession	*N*	Weighted kappa	95% CI
ICU physician/ICU physician	785	0.87	0.85 to 0.89
ICU nurse/ICU nurse	57	0.92	0.87 to 0.97
Research staff/research staff	276	0.84	0.80 to 0.87
ICU nurse/ICU physician	162	0.77	0.71 to 0.83
ICU physician/ICU nurse	309	0.80	0.77 to 0.84
Group: information source			
Patient/patient	394	0.85	0.82–0.88
Family/family	818	0.89	0.87–0.90
Hospital records/hospital records	187	0.89	0.85–0.92
Group: countries			
England	397	0.90	0.87 to 0.97
France	257	0.81	0.78 to 0.85
Spain	186	0.89	0.85 to 0.93
Poland	163	0.89	0.85 to 0.92
Greece	133	0.89	0.85 to 0.94
Germany	125	0.81	0.75 to 0.86
Norway	110	0.71	0.63 to 0.78
Portugal	106	0.82	0.76 to 0.89

We also performed a sensitivity analysis looking at two subgroups according to rater 1: frail (CFS > 4) versus non-frail (CFS < 5). In the frail the kappa was 0.70 (95% CI 0.66–0.74) compared to 0.76 (95% CI 0.74–0.79).

## Discussion

In this large prospective study on frailty assessment in the ICU using the CFS, we found the overall agreement of inter-rater variability in patients > 80 years to be very good. We revealed, however, distinct variations between groups of raters and between countries. The agreement between obtaining CFS from hospital records or family was nearly identical but was lower when the patients were used as the primary source of information.

Frailty is important in order to understand critical ill patients, particularly in advanced age [16], and most studies have demonstrated a close link between frailty and survival. Hence, knowledge of frailty status could be important when issues such as ICU triage and limitation of life-sustaining therapy are discussed. Recent guidelines propose the use of frailty assessment as a part of the triage process to be used with COVID-19 [17]. However, use of frailty in triage setting has its limitations, and is at present not confirmed in prospective studies. However, use of CFS would be effective in analysing the clinical decision-making process of an ICU team. There are several methods to assess frailty, and CFS is frequently used in clinical studies with ICU patients [2] as well as in emergency admission [18] and is also used in routine clinical use in intensive care units outside study settings [19].

Using an instrument such as a frailty score requires knowledge about its performance and with special attention to reliability and construct validity [20]. Of interest is also its ability to predict risk for death, where CFS have been found to perform well. This was recently confirmed in the VIP-2 study, where CFS alone had similar predictive value for 30-day mortality as a model incorporating cognition and functional disability [12], again providing good criterion validity.

The aim of the present study was to document several unanswered questions using CFS. What is the inter-rater variability when analysing more heterogeneous groups of raters using a various source of data for the score? Both aspects are important properties of a clinical test or score. The variation of a score in the same patient between two raters is the inter-rater variability. Overall, our data proves a very high degree of agreement between raters with a weighted kappa of 0.86. Since we had a large number of pairs to study, we could study results in subgroups, both between raters from different professions, the source of information as well as performance across countries. There seems to be better agreement when the raters are from the same profession; physicians or nurses. When the raters are from different professions the agreement is slightly less. We also provide data showing that obtaining information from family members and care-givers or from written records in order to classify CFS is in fact, better compared to the information obtained from the patient. This may have a simple explanation that many elderly patients, although seemingly awake and co-operable, may not perform at their best at the time of ICU admission. Hence important information may not be revealed for the rater. We have found that it can be a problem to differentiate between CFS 4 and 5. This could be important since 5 is the first stage on the frail part of the CFS and 4 is borderline. Recently the CFS was upgraded to version 2.0 and a more detailed guideline in how to understand and use the different levels in the scale have been published [21].

Our study is in line with three recent studies of the inter-rater variability of the CFS. All studies are from single countries with a smaller number of pairs included, and only the overall inter-rater variability was reported. In a study from Canada involving two ICUs [8], different assessors from a research coordinator, an occupational therapist, and a geriatric resident, performed CFS scores in 150 newly admitted ICU patients. They reported no significant differences between the three raters using Spearman’s rank correlation coefficient. In a more recent study from six ICUs in Wales and Scotland, 101 patients were studied with two independent CFS assessments of frailty by assessors from medical or nursing backgrounds [7]. They found a good agreement with a weighted kappa of 0.74 between raters, and also that agreement differed slightly depending on the assessor’s background.

A more recent study comparing CFS scored in 158 adult ICU patients scored by geriatricians and intensivists reported however a poor agreement between raters [9]. The authors suggest that these two groups have a different conception of how frailty presents in critical ill patients as an explanation for this result.

Our study has its limitation: this was not a controlled trial with regard to the choice of profession and source of data used and may have been at the centres' discretion. We also have no information about the clinical experience of the raters nor their age. The study has also strengths. It has a very large sample size of nearly 2000 pairs of raters, with at least three important sources of variation: the profession of raters, source of information, and country.

## Conclusion

In very elderly ICU patients, the CFS has a high compliance rate and exhibits high overall inter-rater agreement with a weighted kappa analysis of 0.86. Furthermore, there are minor variations in performance across different health care professionals, countries and source of data. We found the best agreement using raters from the same health care professionals, but with no difference between pair of nurses or physicians. To determine CFS, caution should be used to rely on the elderly ICU patients as the sole source of information.

Frailty assessment should be routine in the critical ill elderly patients, and the CFS is a good instrument in this respect, and will give a more holistic impression of the patient´s condition prior to admission.

## Supplementary Information

**Additional file 1.** Guideline for reporting of reliability and agreement studies (GRAAS).

**Additional file 2.** List of local investigators.

## Data Availability

Data are embedded in the main database, and as such cannot be opened for general inspection. The main author can at request give an extract from the database for each pair of raters with necessary information about the inter-rater agreement.

## Abbreviations

CFS: Clinical frailty scale
VIP: Very old intensive care patients
ADL: Activity of daily life
IQCODE: Informant questionnaire on cognitive decline in the elderly
GRAAS: Guidelines for reporting of reliability and agreement studies
