# Supplementary material for: Reliability of the Clinical Frailty Scale in very elderly ICU patients: a prospective European study
Source: Ann Intensive Care. 2021 Feb 3;11:22. doi: 10.1186/s13613-021-00815-7 (PMC7856616; doi:10.1186/s13613-021-00815-7)
Supplement: Supplementary file 1 — Additional file 1. Guideline for reporting of reliability and agreement studies (GRAAS). [file 13613_2021_815_MOESM1_ESM.docx]

Reliability of the Clinical Frailty Scale, a prospective study within VIP-2 study INTRODUCTION

**Answers (A) according to the GRRAS guideline**

1. Identify in title or abstract that interrater/intrarater reliability or agreement was investigated.
A: OK

2. Name and describe the diagnostic or measurement device of interest explicitly.
A: OK

3. Specify the subject population of interest.
A: OK

4. Specify the rater population of interest (if applicable).
A: OK

5. Describe what is already known about reliability and agreement and provide a rationale for the study (if applicable).
A: OK

6. Explain how the sample size was chosen. State the determined number of raters, subjects/objects, and replicate observations.
A: No sample size or pre-defined number of raters or subjects. There were no replicate observations

7. Describe the sampling method.
A: OK

8. Describe the measurement/rating process (e.g. time interval between repeated measurements, availability of clinical information, blinding).
A: OK

9. State whether measurements/ratings were conducted independently.
A: OK

10. Describe the statistical analysis.
A: OK

11. State the actual number of raters and subjects/objects which were included and the number of replicate observations which were conducted.
A: OK pts, no numbers of raters

12. Describe the sample characteristics of raters and subjects (e.g. training, experience).
A: OK subjects, partially raters

13. Report estimates of reliability and agreement including measures of statistical uncertainty.
A: OK

14. Discuss the practical relevance of results.
A: OK

15. Provide detailed results if possible (e.g. online)
A: Done in the manuscript

**GRAAS guideline:**

Kottner J, Audigé L, Brorson S, Donner A, Gajeweski BJ, Hróbjartsson A, Robersts C, Shoukri M, Streiner DL. Guidelines for reporting reliability and agreement studies (GRRAS) were proposed.

J Clin Epidemiol.  2011;64(1):96-106 PMID: [21130355](http://www.ncbi.nlm.nih.gov/pubmed/21130355)
