# Supplementary material for: Reliability of the Clinical Frailty Scale in very elderly ICU patients: a prospective European study
Source: Ann Intensive Care. 2021 Feb 3;11:22. doi: 10.1186/s13613-021-00815-7 (PMC7856616; doi:10.1186/s13613-021-00815-7)
Supplement: Supplementary file 2 — Additional file 2. List of local investigators. [file 13613_2021_815_MOESM2_ESM.pdf]

**Austria**				
**AT**	Michael Joannidis	Medical University Innsbruck	Division Of Intensive Care And Emergency Medicine,	Innsbruc
**AT**	Philipp Eller	Medical University Graz	Allgemeine Medizin Intensivstation	Graz
**AT**	Raimund Helbok	Medical University Of Innsbruck	Department Of Neurology, Neurocritical Care Unit	Innsbruc
**AT**	René Schmutz	Hospital Of St. John Of God	Icu B5	Wienna
**Denmark**				
**DK**	Anders Bastiansen	Bispebjerg Hospital	Intensiv Terapi Afsnit	Copenha
**DK**	Christoffer Sølling	Viborg Regional Hospital	Department Of Anaesthesiology And Intensive Care	Viborg
**DK**	Thomas Elkmann	Aarhus University Hospital	Intensive Care North	Aarhus
**England (GB)**				
**EN**	Agnieszka Kubisz-Pudelko	Yeovil District Hospital	Intensive Care Unit	Yeovil
**EN**	Alan Pope	Peterborough City Hospital	Critical Care Unit	Petersbo
**EN**	Amy Collins	Queen Elizabeth Hospital	Critical Care Queen Elizabeth Hospital	
**EN**	Ashok S. Raj	Croydon University Hospital	Croydon University Hospital Itu	Croydon
**EN**	Carole Boulanger	Royal Devon & Exeter Nhs Foundation Trust	Intensive Care Unit	Exeter
**EN**	Ciaran Hart	Maidstone	Intensive Care/high Dependency	Maidston
**EN**	Clare Bolger	University Hospital Southampton	General Intensive Care Unit	Southam
**EN**	Georgina Randell	Norfolk And Norwich University Hospital	Critical Care Complex	Norwich
**EN**	Ingeborg D Welters	Royal Liverpool University Hospital	Intensive Care	Liverpool
**EN**	Jason Cupitt	Blackpool Victoria Hospital	General Critical Care Unit	Blackpoo
**EN**	Jeremy Henning	James Cook University Hospital	Icu 2 And 2	Midlesbr
**EN**	Joanne Jones	Tunbridge Wells Hospital	Intensive Care Unit	Pembury
**EN**	Kiran Salaunkey	Royal Papworth Hospital Nhs Foundation Trust	Icu	Cambrid
**EN**	Laura Ortiz-Ruiz De Gordoa	Royal Sussex County Hospital	Level 7	Brighton

**EN**	Madhu Balasubramaniam	Royal Bolton Hospital	Royal Bolton Critical Care	Bolton
**EN**	Marcela Vizcaychipi	Chelsea And Westminster Hospital	Adult Intensive Care Unit	London
**EN**	McDonald Mupudzi	Hampshire Hospitals Foundation Trust	Basingstoke And North Hampshire Hospital	Basingst
**EN**	Michael Reay	Russells Hall Hospital	Intensive Care Unit Russells Hall	Dudley
**EN**	Michael Spivey	Royal Cornwall Hospital Trust	Critical Care Unit	Cornwall
**EN**	Nick Spittle Nick Spittle	Chesterfield Royal Hospital	Intensive Care Unit	Chesterfi
**EN**	Nigel White	Royal Bournemouth Hospital	Bournemouth Critical Care Unit	Bournem
**EN**	Patricia Williams	Dorset County	Icu Dch	Dorchest
**EN**	Patrick Morgan	Surrey And Sussex Healthcare Nhs Trust	East Surrey Hospital	Redhill
**EN**	Rachel Savine	Royal Surrey County Hospital	Royal Surrey	Guildford
**EN**	Reni Jacob	Wirral University Teaching Hospital	Critical Care	Birkenhe
**EN**	Richard Innes	Musgrove Park Hospital	Critical Care Unit	Taunton
**EN**	Tarkeshwari Mane	North Tees University Hospital	Critical Care Unit	Stockton
**EN**	Waqas Khaliq	University Hospital Lewisham	Icu/hdu Lewisham	Lewishar
**France**				
**FR**	Bertrand Guidet	Saint Antoine	Medecine Intensive Reanimation	Paris
**FR**	Didier Thevenin	Ch Dr Schaffner	Reanimation Polyvalente	Lens
**FR**	Guillaume Savary	Hôpital Cochin	Médecine Intensive Réanimation	Paris
**FR**	Hervé Mentec	Victor Dupouy	Service De Réanimation Polyvalente Et Usc	Argenteu
**FR**	Jean-Philippe Rigaud	Dieppe General Hospital	Médecine Intensive Réanimation	Dieppe
**FR**	Julien Maizel	Chu Amiens	Reanimaiton Medicale	Amiens
**FR**	Kelly Tiercelet	Groupe Hospitalier Paris Saint Joseph	Réanimation Polyvalente	Paris
**FR**	Maud Mousset Hovaere	Ch Dax	Réanimation Polyvalente	Dax
**FR**	Nathalie Rolin	Groupe Hospitalier Sud Ile De France	Département De Médecine Intensive	Melun
**FR**	Philippe Burtin	Clinique Du Millenaire	Reanimation Chirurugicale Ii Et Iii	Montpelli

**FR**	Saad Nseir	Chu Lille	Critical Care Center	Lille
**Germany**				
**DE**	Christian Rabe	Klinikum Rechts Der Isar Tu München	Toxikologische Intensivstation	München
**DE**	Eberhard Barth	University Hospital Ulm	Anesthesiologic Intensive Care Department	Ulm
**DE**	Kristina Fuest	Klinikum Rechts Der Isar, School Of Medicine,	Intensivstation Is2/l2a	München
**DE**	Michael Schuster	Universitätsmedizin Der Johannes Gutenberg-	Anästhesie-intensivstation	Mainz
**DE**	Patrick Meybohm	University Hospital Frankfurt	Department Of Anaesthesiology, Intensive Care Medicine And	Frankfurt
**DE**	Sebastian Allgäuer	Robert-bosch-krankenhaus	1d	Stuttgart
**DE**	Stefan J Schaller	Klinikum Rechts Der Isar, School Of Medicine,	Intensivstation Is1 / M2b	München
**DE**	Stefan Schering	University Hospital Leipzig	Department Of Anesthesiology And Intensive Care Medicine	Leipzig
**DE**	Stephan Steiner	St Vincenz Hospital	Intensive Care Unit	Limburg/l
**DE**	Tobias Graf	Universitätsklinikum Schleswig-holstein	Iki 12a	Lübeck
**Greece**				
**GR**	Aristeidis Vakalos	Xanthi General Hospital	Xanthi Icu	Xanthi
**GR**	Elli Niki Flioni	General Hospital Agios Pavlos	Icu Agios Pavlos	Thessalo
**GR**	Evangelia Neou	General Hospital Of Larissa	General Icu	Larissa
**GR**	Georgios Papathanakos	University Hospital Of Ioannina	Intensive Care Unit	Ioannina
**GR**	Ioannis Koutsodimitropoulos	General Hospital Of Eleusis Thriassio	Icu Latsio	Eleusis
**GR**	Kounougeri Aikaterini	Konstantopouleion Gen. Hospital	General Icu	Athens
**GR**	Nikoletta Rovina	Sotiria Hospital	Icu 1st Department Of Pulmonary Medicine Athens Medical	Athens
**GR**	Stylliani Kourelea	General Hospital Of Patra	Icu	Achaia
**GR**	Vasiiios Zidianakis	Agioi Anargiroi Hospital	General Icu	Athens
**GR**	Vryza Konstantinia	Theagenio	Meth Theagenio	Theassal
**GR**	Zoi Aidoni	University General Hospital Ahepa	Metha	Thessalo

**Ireland**				
**IE**	Brian Marsh	Mater Misericordiae University Hospital	Department Of Critical Care Medicine	Dublin
**IE**	Catherine Motherway	University Hospital Limerick	Uhl Icu	Limerick
**IE**	Chris Read	University Hospital Galway	General Icu	Galway
**Italy**				
**IT**	Andrea Neville Cracchiolo	Arnas Ospedale Civico De Christina Benfratelli	Terapia Intensiva Polivalente Con Trauma Center	Palermo
**IT**	Aristide Morigi	Istituto Ortopedico Rizzoli	Tipo	Bologna
**IT**	Italo Calamai	San Giuseppe	Terapia Intensiva	Empoli
**IT**	Stefania Brusa	Humanitas Reseach Hospital	General Icu	Milan
**Libya**				
**LY**	Wesal Ali Belkhair	Tripoli Medical Center	Ccu	Tripoli
**Netherlands**				
**NL**	Alexander D. Cornet	Medisch Spectrum Twente	Intensive Care Center	Ensched[illegible]
**NL**	Lenneke Haas	Diakonessenhuis Utrecht	Intensive Care	Utrecht
**NL**	Oscar Hoiting	Canisius Wilhelmina Ziekenhuis	C38	Nijmegen
**NL**	Rik T. Gerritsen	Medical Centre Leeuwarden	Department Of Intensive Care	Leeuwar[illegible]
**Norway**				
**NO**	Bente Jannestad	Sykehuset Telemark	Intensiv Skien	Skien
**NO**	Britt Sjøbøe	Haukeland University Hospital	General Icu	Bergen
**NO**	Eva Rice	Ålesund	Medisinsk Intensiv	Ålesund
**NO**	Finn H. Andersen	Ålesund Hospital	Dept. Anesthesia And Intensive Care, Surgical Icu	Ålesund
**NO**	Hans Frank Strietzel	Kristiansund Sykehus Helse Møre Og Romsdal Hf	Intensiv Kristiansund	Kristians[illegible]
**NO**	Jørund Langørgen	Haukeland University Hospital	Medisinsk Intensiv Og Overvåkning (mio)	Bergen
**NO**	Kirsti Tøien	Oslo University Hospital	Intensive Care Section Ullevaal	Oslo

**NO**	Kristian Strand	Stavanger University Hospital	Department Of Intensive Care	Stavange[illegible]
**Poland**				
**PO**	Anna Kluzik	Heliodor Swiecicki Clinical Hospital At The Karol	Anaesthesiology Intensive Care And Pain Treatment	Poznań
**PO**	Dariusz Maciejewski	Regional Teaching Hospital	Department Of Anaesthesiology And Intensive Care	Bielsko-b[illegible]
**PO**	Lukasz J. Krzych	University Clinical Center Katowice	Department Of Anaesthesiology And Intensive Care - School	Katowice
**PO**	Maciej Zukowski	Teching Hospital No 2	Department Anaesthesiology Intensive Therapy And Acute	Szczecin
**PO**	Małgorzata Lipińska-Gediga	4th Military Hospital In Wrocław	Anesthesia And Intensive Care Unit	Wrocław
**PO**	Marta Serwa	Central Clinical Hospital Ckd - University Medical	Anaesthesia And Intensive Care Clinic	Lodz
**PO**	Miroslaw Czuczwar	First Independent Teaching Hospital No. 1	Ii Department Of Anesthesiology And Intensive Care	Lublin
**PO**	Mirosław Ziętkiewicz	Krakowski Szpital Specjalistyczny Im. Jana Pawła	Thoracic Anaesthesia And Respiratory Icu	Kraków
**PO**	Natalia Kozera	Wroclaw Medical University	Department Of Anesthesiology And Intensve Therapy	Wroclaw
**PO**	Paweł Zatorski	Infant Jesus Teaching Hospital	I Department Of Anaesthesiology And Intensive Care	Warsaw
**PO**	Piotr Galkin	Regional Hospital In Bialystok	Department Of Anaesthesiology And Intensive Care	Bialystok
**Portugal**				
**PT**	Ana Margarida Fernandes	Hospital S. José, Chulc Epe	Uci Neurocríticos E Trauma	Lisboa
**PT**	Ana Rita Santos	Hospital São Francisco Xavier	Unidade Cuidados Intensivos Polivalente	Lisbon
**PT**	Cristina Sousa	Hospital Da Luz	Uci Hospital Da Luz	Lisboa
**PT**	Inês Barros	Hospital De Viseu	Ucip	Viseu
**PT**	Isabel Amorim Ferreira	Hospital Professor Doutor Fernando Fonseca Epe	Serviço De Medicina Intensiva Smi	Amadora
**PT**	Jacobo Bacariza Blanco	Hospital Garcia De Orta - Hgo	Serviço De Medicina Intensiva	Almada
**PT**	Jose Maia	Centro Hospitalar De Trás Montes E Alto Douro	Serviço De Medicina Intensiva	Vila Real
**PT**	Nuno Catorze	Chmt-abrantes	Smi	Abrantes
**Russian**				
**RU**	Vladislav Belskiy	Privolzhskiy District Medical Center	Department Of Anesthesiology And Intensive Care	Nizhniy N[illegible]

**Spain**				
**ES**	Africa Lores	Hospital De Bellvitge	Uci	Barcelon
**ES**	Catia Cilloniz	Hospital Clinic Of Barcelona	Respiratory Intensive Care Unit	Barcelon
**ES**	David Perez-Torres	Hospital Universitario Río Hortega	Uvi Polivalente Y Coronaria	Valladoli
**ES**	Emilio Maseda	Universitario La Paz	Surgical Icu	Madrid
**ES**	Enver Rodriguez	General Universitario De Castellón	Servicio De Medicina Intensiva	Castelló
**ES**	Estefania Prol-Silva	Hospital Universitario Río Hortega	Uvi Neurocríticos Trauma Y Quemados	Valladoli
**ES**	Gaspar Eixarch	Hospital De Tortosa Verge De La Cinta	Servei De Medicina Intensiva	Tortosa
**ES**	Gerardo Aguilar	Clínico Universitario De Valencia	Surgical Intensive Care Unit	Valencia
**ES**	Marián Irazábal Jaimes	Hospital General De Catalunya	Hgc	Barcelon
**ES**	Mercedes Ibarz Villamayor	Hospital Universitario Sagrado Corazon	Intensive Care Unit	Barcelon
**ES**	Patricia Jimeno Cubero	Complejo Hospitalario De Segovia	Icu Segovia	Segovia
**ES**	Teresa Tomasa	Germans Trias I Pujol Hospital	General Icu	Badalon
**Sweden**				
**SE**	Camilla Brorsson	Umeå University	Department Of Surgical And Perioperative Sciences,	Umeå
**SE**	Jessica Nauska	Blekingesjukhuset	Intensivvårdsavdelning 31	Karlskron
**SE**	Joakim Sivik	Alingsås Lasarett	Intensivvårdsavdelningen	Alingsås
**SE**	Lina De Geer	Linköping University Hospital	Icu Linköping	Linköpin
**SE**	Sten Walther	Linköping University Hospital	Cardiothoracic Intensive Care Unit	Linköpin
**Switzerland**				
**CH**	Filippo Boroli	Hopitaux Universitaires De Genève	Adult Intensive Care Unit	Geneva
**CH**	Joerg C. Schefold	University Of Bern Inselspital	Department Of Intensive Care Medicine	Bern
**Turkey**				
**TR**	Ismail Yıldız	Ordu University Training And Research Hospital	General Icu	Ordu

**Ukraine**				
**UA**	Ihor Yovenko	Dnipro Mechnikov Regional Clinical Hospital	Intensive Care Unit Of Polytrauma	Dnipro
**UA**	Yuriy Nalapko	European Wellness Academy, Luhansk Regional	Icu 1	Lugansk
**UA**	Yuriy Nalapko	European Wellness Academy, Luhansk Regional	Icu 2	Lugansk
**Wales (GB)**				
**WL**	Richard Pugh	Glan Clwyd Hospital	Critical Care	Bodelwy[illegible]
